# Assessment of endodontically treated teeth's periapical bone loss using CBCT before and after orthodontic treatment

**DOI:** 10.6026/973206300213292

**Published:** 2025-09-30

**Authors:** Subhrajeet Narayan Sahoo, Bejoy P.U., Yogendra Kumar Singh, Sajid Ahmed Sanadi, Rishav Singh, Richa Goel, Abhigyan Manas

**Affiliations:** 1 Department of Orthodontics, Institute of Dental Sciences, Siksha O Anusandhan University, Bhubaneswar, Odisha, India; 2 Department of Orthodontics and Dentofacial Orthopedics, Malabar Dental College & Research Centre, Malappuram, Kerala-679578, India; 3 Department of Dentistry, Government Medical College, Azamgarh, Uttar Pradesh, India; 4 Department of Oral and Maxillofacial Surgeon, King Abdullah Medical City, Makkah Saudi Arabia; 5 Department of Pedodontics & Preventive Dentistry, Maharaja Ganga Singh Dental College & Research Centre, Sri Ganganagar, Rajasthan, India; 6 Department of Public Health Dentistry, Karnavati School of Dentistry, Karnavathi University, Gandhinagar, Gujarat, India; 7 Department of Oral and Maxillofacial Surgery, Faculty of Dental Sciences, Uttar Pradesh University of Medical Sciences, Saifai, Etwash, Uttar Pradesh, India

**Keywords:** Bone Loss, CBCT, endodotic, orthodontic treatment, periodontal

## Abstract

Fixed orthodontic treatment has impact on periapical bone and tissue healing. Therefore, it is of interest to evaluate the endodontically treated teeth's loss of periodontal bone both before and after orthodontic treatment. Total 100 participants underwent for fixed orthodontic treatment with bone loss around endodontically treated teeth (ETT) with cone beam computed tomography (CBCT) images were recruited for the study. The periapical changes were assessed clinically and radiographically. Following orthodontic treatment, the extent of the alveolar bone surrounding ETT decreased, as did the extent of the periapical lesion.

## Background:

Preventing apical periodontitis is the aim of endodontic therapy. Endodontic treatment aims to eradicate infection because apical periodontitis is an infectious disease. Proper mechanical and chemical disinfection eliminates microorganisms. In addition to reducing inflammation, root canal obturation and irritant elimination promote the healing of the periapical lesion [1]. Root canal obturation (RCT) and irritant removal both lessen inflammation and encourage the repair of the periapical disease [2]. This further encourages bone repair in the wounded periapical area. A sign of periapical alterations is bone regeneration [3]. Identification of the periapical alterations is aided by radiographic assessment [4]. Alveolar bone resorbs and restructures as a result of the forces placed on the periapical region during orthodontic tooth movement. Similar to osteoclastic activity in inflammatory reactions, alveolar bone resorption manifests as an inflammatory response. The direction of the applied orthodontic treatment force is where resorption takes place [5, 6]. Orthodontic treatment may have an effect on pre-existing lesions in patients who have undergone endodontic treatments. Due to periapical inflammation, orthodontic application (OA) may alter pre-existing periapical lesions (PL) [7]. In addition to root resorption, orthodontic therapy may result in changes to pre-existing periapical lesions because of this inflammatory response [8]. The reported article on impact of using orthodontic pressure on the periapical radiolucency of endodontic treated teeth is very scares. Therefore, it is of interest to assess the periapical radiolucency of endodontically treated teeth using CBCT both before and after orthodontic therapy.

## Materials and Methods:

This *in vitro* retrospective research was done in the department of Orthodontics. Orthdodontically treated patients records were obtained from the department of Orthodontics from 2016 to 2024. Total 100 treated cases underwent fixed orthodontic treatment with bone loss around endodotically treated teeth (ETT) were recruited for the study. Patients who received root canal therapy and whose CBCT scans were available before and after orthodontic treatment with a gap of more than a year between them were included. Endodotically treated teeth (ETT) include endoontic treatment to permanent incisors, premolar and molars of both jaws. Patients who had orthodontic tooth extractions or primary teeth were not included. Information about the size of the periapical lesion (PL), the number of ETTs, their placement, the length of orthodontic therapy, and demographics were taken into account. Assessment was done for changes in periapical lesion (PL), periapical radiolucency (PR) size around ETT, dimension of alveolar bone in buccolingual direction around ETT. CBCT was used to assess the size of the endodontically treated tooth's periapical lesion (SPL). The most recent CBCT photos following orthodontic treatment as well as pre-orthodontic therapy CBCT images were examined. The obtained data was statistically evaluated using Statistical Package for Social Sciences (SPSS) version 25 (IBM, Chicago, USA) with One way ANOVA and student t test at P<0.05.

## Results:

Table 1 (see PDF) indicates that, there was a reduced in the size of periapical lesions (SPL) of 54.1% while increase lesion size is 45.9% of lesions. It was found that generally there was a reduction in the size of PL after orthodontics treatment, however, the findings were non statistically considerable (P = 0.267). Total 100 participants had 120 root canal treated teeth with periapical lesions. In ETT, the alveolar bone's dimensions decreased in the SI, BL, and MD directions. Table 2 (see PDF) shows that the results were statistically considerable.

## Discussion:

It has long been recognised that orthodontic and endodontic treatments are related. The teeth and surrounding tissues may be affected in a number of ways by endodontic treatment during orthodontic procedures [9]. The alveolar bone loss and SPL surrounding ETT were evaluated in the current CBCT study both before and after orthodontic therapy. Prasad *et al.* evaluated the periapical radiolucency (PR) and alveolar bone loss surrounding endodontic treated teeth (ETT) prior to and following orthodontic treatment. They came to the conclusion that following orthodontic treatment, the size of the periapical lesion surrounding ETT had decreased [4]. Our findings are consistent with the results. According to a study by Hajihassani *et al.* mandibular molars that had endodontic treatment displayed greater lesions in the furcation area and considerably larger apical lesions compared to maxillary molars [10]. By curing the periapical lesions, the quality of endodontic therapy in the Eckerbom *et al.* trial demonstrated a more pronounced decrease in SPR [11]. Kim *et al.* use cone-beam computed tomography (CBCT) to evaluate the periapical radiolucency of endodontically treated teeth both before and after orthodontic therapy. He proposed that the radiolucency will heal more fully the longer the follow-up time. Additionally, the extent of the periapical radiolucency (SPR) was not significantly impacted by the patient's age. They came to the conclusion that the alterations in the SPR in teeth that had received endodontic therapy were not significantly impacted by orthodontic treatment [8]. According to Ianos *et al.* endodontically treated teeth with poor filling quality have a significantly higher risk of periapical bone destruction after orthodontic treatment, and the periapical bone destruction probability index (PRI) showed a significant increase in periapical destruction after orthodontic treatment [9]. When it comes to identifying and quantifying periapical lesions, CBCT pictures are more precise than periapical and panoramic radiographs [12, 13]. Tsai *et al.* used CBCT to estimate the diameter of periapical lesions, and when the diameter was greater than 0.8 mm, the results demonstrated high accuracy [14]. One typical side effect of orthodontic treatment is root resorption [5]. Due to inflammation in the periapical lesion, root resorption begins with orthodontic pressures and develops similarly to bone resorption [8]. Apical root resorption (ARR), which resembles bone disintegration, can result with orthodontic therapy due to the periapical tissue's inflammatory response. According to Liu *et al.* endodontically treated teeth had much less orthodontically induced root resorption; however, there are important elements that affect this, including the type of therapy, the appliance used, the length of treatment, and the distance between the roots [15]. The Results of the RCT showed no significant correlation with the orthodontic traction distance or rotation angle [16].

## Conclusion:

The size of the alveolar bone and periapical lesions surrounding endodontically treated teeth decreased following orthodontic treatment.
